# Design and Evaluation of a Portable Pinhole SPECT System for ^177^Lu Imaging: Monte Carlo Simulations and Experimental Study

**DOI:** 10.3390/diagnostics15111387

**Published:** 2025-05-30

**Authors:** Georgios Savvidis, Vasileios Eleftheriadis, Valentina Paneta, Eleftherios Fysikopoulos, Maria Georgiou, Efthimis Lamprou, Sofia Lagoumtzi, George Loudos, Paraskevi Katsakiori, George C. Kagadis, Panagiotis Papadimitroulas

**Affiliations:** 1 BIOEMTECH, 15344 Athens, Greece; savvidisgeorgios@bioemtech.com (G.S.); mgeorgiou@bioemtech.com (M.G.); george@bioemtech.com (G.L.); 2 3dmi Research Group, Department of Medical Physics, University of Patras, 265504 Rion, Greecegkagad@gmail.com (G.C.K.); 3 Medical Informatics Laboratory, Department of Medicine, University of Thessaly, 41500 Larissa, Greece

**Keywords:** pinhole collimator, lutetium, MC simulations, GATE, SPECT

## Abstract

**Background**/**Objectives:** Lutetium-177 is a widely used radioisotope in targeted radionuclide therapy, particularly for treating certain types of cancers relying on beta and low-energy gamma emissions, making it suitable for both therapeutic and post-therapy monitoring purposes. The purpose of this study was to evaluate the technical parameters for developing a prototype portable gamma camera dedicated to ^177^Lu imaging applications. **Methods**: The well-validated GATE Monte Carlo toolkit was used to study the characteristics of the system and evaluate its performance in terms of spatial resolution, sensitivity, and image quality. For this purpose, a series of Monte Carlo simulations were executed, modeling a channel-edge aperture pinhole collimator incorporating a variety of computational phantoms. The final configuration of the prototype was standardized, incorporating the crystal size, collimator design, shielding, and the optimal FOV. After the development of the actual prototype camera, the system was also validated experimentally on the same setups as the simulations. **Results**: The final configuration of the prototype imaging system was standardized based on simulation results and then experimentally validated using physical phantoms under equivalent conditions. A minification of 1:5, spatial resolution of 1.0 cm, and sensitivity of 5.2 Cps/MBq at 10 cm distance source-to-collimator distance were assessed and confirmed. The experimental results agreed within 5% of simulated values. **Conclusions**: This study establishes the technical feasibility and foundational performance of a portable pinhole imaging system for potential clinical use in ^177^Lu imaging workflows and thereby improving therapeutic effectiveness.

## 1. Introduction

Lutetium-177 (^177^Lu) is a versatile radioisotope with significant clinical importance in both molecular radiotherapy (MRT) and imaging diagnostics. Its ability to deliver targeted radiation therapy while allowing for concurrent imaging makes it an invaluable tool in the treatment of certain cancers, particularly neuroendocrine tumors (NET) and prostate cancer [1,2,3,4,5]. To translate the therapeutic benefits of ^177^Lu observed in clinical trials into broader clinical practice, accurate organ-level dose assessment is essential for optimizing treatment outcomes [1,4,6]. Personalized dosimetry is a critical component of ^177^Lu-based therapy optimization, as it relies on quantitative imaging to accurately characterize the in vivo biodistribution and biokinetics of the administered radiopharmaceutical [6,7,8].

Due to the importance of optimizing imaging techniques to enhance treatment, numerous studies have focused on improving these imaging methods. The optimized techniques are based on collimator designs, scatter correction methods, and the evaluation of various iterative reconstruction algorithms to achieve visual assessments of the produced images [9,10,11]. However, limitations still exist in post-therapy image acquisition. Toderas et al. mention that the role of ^177^Lu SPECT/CT imaging after therapy is not well established [12]. Additionally, studies have shown that to further improve the assessment of ^177^Lu post-therapy dosimetry based on multiple time points is more accurate and suitable for routine clinical implementation [13]. Recently, hand-held cameras have been developed for ^177^Lu imaging [14], for small animal SPECT imaging [15], and for small field-of-view applications in nuclear medicine [16]. Moreover, multiple investigations have been focused on establishing gamma camera calibration factors specific to ^177^Lu imaging [17,18]. Such a portable camera could be used as an additional tool in clinical practice to facilitate following the patient in vivo distribution of ^177^Lu during therapy in the patient and being able to reconsider the treatment plan for the subsequent injections.

Given the limitations in both preclinical and clinical settings, the aim of the present study was to develop an imaging device, based on a pinhole collimator, dedicated to ^177^Lu applications. In preclinical imaging, pinhole collimators are predominantly used in many studies [19,20] to achieve magnification and high spatial resolution for imaging small organs and structures while recently several studies have investigated the multi-pinhole collimators concept as well [21,22,23,24]. This technique is particularly useful for research involving small animals, such as mice and rats, where detailed visualization of tiny anatomical features is essential. In addition, magnification techniques are also used in clinical imaging applications using probes and dedicated cameras for imaging specific organs [25,26,27].

In this study, we developed an innovative, cost-effective clinical device incorporating a single-pinhole collimator design to achieve minification, enabling coverage of a larger patient area. To ensure ease of use and support bedside patient imaging, the system was designed to be portable, facilitating the acquisition of follow-up images after treatment. The device is intended to assist clinicians by providing qualitative, post-therapy imaging of ^177^Lu distribution, supporting treatment monitoring.

To evaluate and validate the characteristics of the proposed camera, several simulations were performed with geometrical phantoms as well as clinical and preclinical data. The concept of the proposed camera was based on minifying the field of view (FOV) in a small detector allowing multiple bedside imaging acquisitions. Prior to the construction of the actual system and the final assessment of the performance, the in silico evaluation of the device was conducted in the GATE v9.1 Monte Carlo (MC) toolkit [28,29,30], and several parameters had been tested for optimization purposes.

## 2. Materials and Methods

### 2.1. MC Simulations

**Figure 1:**
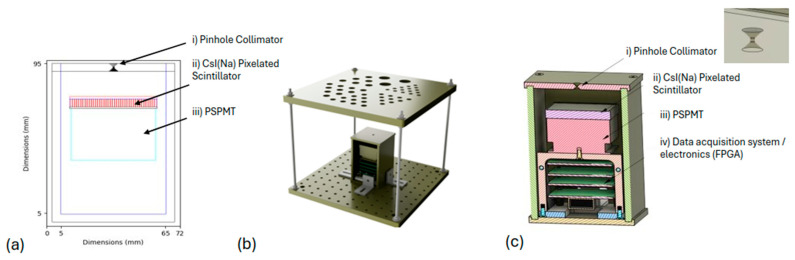
The proposed portable camera: (**a**) GATE model, (**b**) prototype camera and Derenzo-like phantom, and (**c**) cross-sectional view of the prototype camera.

MC simulations were executed to standardize the system prior to its construction, using the well-validated GATE v9.1 toolkit [31,32]. More precisely, a series of simulations were applied and tested to investigate the technical parameters of the system such as (a) the pinhole-to-detector and pinhole-to-object distances; (b) the pinhole characteristics, i.e., diameter, height, acceptance angle; (c) the scintillator pixel size; and (d) the overall size of the detector and housing. A simulated model of the system is depicted in Figure 1a.

GATE is an open-source MC simulation toolkit based on Geant4 code [33], modeling the radiation transportation and interactions within matter. Specifically, GATE v9.1 was used for the execution of the simulations. The “standard model” (emstandard_opt3) was selected, which is recommended for the electromagnetic physical processes. Moreover, this model is effective and validated at the energy range of 1 keV–100 TeV, which is the range of interest in nuclear medicine procedures. For the simulation of ^177^Lu, the “ion” source type of Geant4 was used to accurately model the whole emission spectrum incorporating its radioactive decay process.

The implementation of simulations was performed in a high-performance computing center (HPC) (Yotta Advanced Computing, Rijeka, Croatia) for speeding up their execution, utilizing 112 parallel jobs and remarkably reducing the computational time.

### 2.2. Portable Gamma Camera

The proposed pinhole gamma camera consists of four main components:

i. Tungsten shielding box and pinhole collimator, Weldstone Components GmbH, Germany [34];
ii. CsI(Na) pixelated scintillator, EPIC Crystals (type: GAGG: Gadolinium Aluminium Gallium Garnet), China [35];
iii. Position sensitive photomultiplier (PSPMT), Hamamatsu, Japan [36];
iv. Data acquisition system/electronics (FPGA) [37].

In the study of Smith and Jaszczak [38] the dependance of the collimator’s acceptance angle and its sensitivity was investigated, providing insights on the appropriate pinhole characteristics for our application. Based on this, the diameter of the pinhole was selected equal to 1.2 mm, with a channel height equal to 5 mm and an acceptance angle equal to 94 degrees. To achieve a minification factor of 5, the object-to-pinhole and detector-to-pinhole distances were 100 mm and 20 mm, respectively.

Specifically, a 50 × 50 mm^2^ channel-edge pinhole gamma camera was modeled within the GATE environment, offering an active FOV of 250 × 250 mm^2^ in a minification configuration. The specifications of the proposed camera are outlined in Table 1, followed by a visual depiction of the developed system in Figure 1b,c.

The goal of the proposed setup was to achieve a minification of the FOV by a factor of 5, with spatial resolution of ~1 cm, in a source-to-collimator distance of 10 cm. Such a configuration will allow a clinically useful small imaging system aiming to detect small structures (larger than 1 cm in diameter).

### 2.3. System’s Evaluation

The system was evaluated in silico, implementing MC simulations, and subsequently experimentally after the development of the actual system. An investigation into the system’s accuracy with different geometrical objects was implemented to study the camera’s spatial resolution, sensitivity, and its overall performance. For all the actual experiments using the protype camera, the injected ^177^Lu activity was mixed with deionized water, and we used a set of different 3D printed or machined phantoms.

To conduct a more thorough evaluation of the proposed system, we launched a complementary investigation in terms of preclinical level using a fillable rat phantom (BIOEMTECH, Athens, Greece) [39] in its digital form for the simulations (digital phantom) and 3D printed (physical phantom) for the actual experiment. Lastly, we conducted 2 clinical simulations (i) with the XCAT anthropomorphic model [40] incorporating a clinical biodistribution of ^177^Lu-DOTATATE [41] and (ii) using clinical ^177^Lu imaging data (SPECT/CT) to further evaluate the system’s performance in clinical applications. The overall set of evaluation simulations and experimental procedures are described in the following sub-sections.

#### 2.3.1. Non-Uniformity Correction

Geometric detector non-uniformity was corrected by normalizing the acquired images with the image of a flood source filled with ^177^Lu solution. More specifically, the values of each pixel in the acquired images were divided by the values of the corresponding pixel of the flood image. For correcting the simulated images accordingly, a 250 × 250 × 10 mm^3^ box (covering the whole active FOV) filled with ^177^Lu solution and total activity of 80 MBq was simulated for a total duration of 90 min to achieve a high statistics image (with low uncertainty). The number of primaries in the simulation was ~4.3 × 10^10^ with an isotropic emission (4π steradian). The final flood image for non-uniformity correction was produced using a ±10% energy window at the peak of 113 keV. For non-uniformity correction in the experimental results, a flood source/phantom with the same dimensions (250 × 250 × 10 mm^3^) was filled with ^177^Lu solution of 37 MBq and placed 100 mm away from the pinhole. Data have been acquired for 12 h to achieve a high statistics flood image, which will be used as a pre-calculated correction factor for the experimental acquisitions.

#### 2.3.2. Derenzo-like Phantom

**Figure 2:**
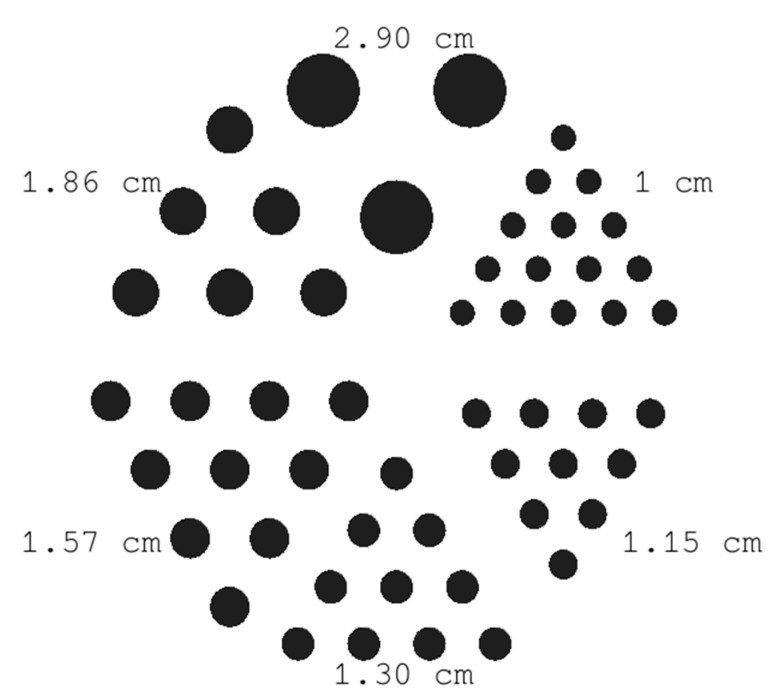
The central slice of the used 3D Derenzo-like phantom. The diameter of each rod is depicted on the corresponding rod group.

To evaluate the spatial resolution, a series of simulations and experiments were conducted using a Derenzo-like phantom [42]. This setup is one of the most common quality control phantoms for nuclear medicine applications, providing a reliable tool for performance assessment and quality assurance in SPECT systems. It has been extensively evaluated in various studies to standardize performance in medical systems [43,44]. The phantom pattern is a series of rods separated by the same distance as their diameter (2.9, 1.86, 1.57, 1.3, 1.15, and 1 cm), assigned with material that mimics the radiodensity of human tissue. In our case, since we aimed to achieve a spatial resolution equal to ~1 cm, we constructed a Derenzo-like phantom with rods of various diameters, as depicted in Figure 2, filled with activity of 18.5 MBq (in each of them). The dimension of the whole phantom was 28.6 × 28.6 × 1.5 cm^3^, and it was placed at a 100 mm distance from the pinhole collimator while it was machined from an acetal plate.

#### 2.3.3. Off-Center Point Sources and Spheres at Different Depths

A variety of geometrical phantoms (setups of point sources) were modeled to further evaluate the system’s performance. Initially, a sphere with a diameter equal to 2 mm was filled with water while a ^177^Lu point source was placed in the middle of the sphere with 1000 MBq activity. The source was placed at the center of the FOV, with the number of primaries being equal to 7 × 10^9^ (the acquisition time was set to 7 s). This simulation (and the respective actual experiment) was repeated with point sources at 5 different positions (offsets from the center), namely at 2 cm, 4 cm, 6 cm, 8 cm, and 10 cm across the FOV, to assess the detector’s sensitivity in the whole range of the FOV [38]. To mimic experimentally a point source, we placed 5 μL of ^177^Lu solution (using deionized water) at the bottom of a standard Eppendorf tube as a standard experimental procedure. Our objective was to measure the detected counts and observe the variability of sensitivity across the horizontal offset of the sources. Sensitivity measurements are essential for describing detector efficiency in nuclear imaging modalities like SPECT, with higher sensitivity values indicating improved image quality. The sensitivity of a detector is defined as the number of the detected events per unit of time from a radioactive source, and it is expressed in counts per second (cps) usually normalized to the source activity (as cps/MBq), as used in this work. Specifically, the number of detected counts (as cps/MBq) has been extracted for each source measurement within a ±35% energy window in both energy peaks (total window at 73–281 keV) and then has been normalized to the maximum value, namely to the one acquired with the source positioned at the center of the FOV (zero offset) and is therefore referred to as normalized sensitivity. To assess the absolute sensitivity value of the system, we used the detected counts from the source placed at the center of the FOV.

**Figure 3:**
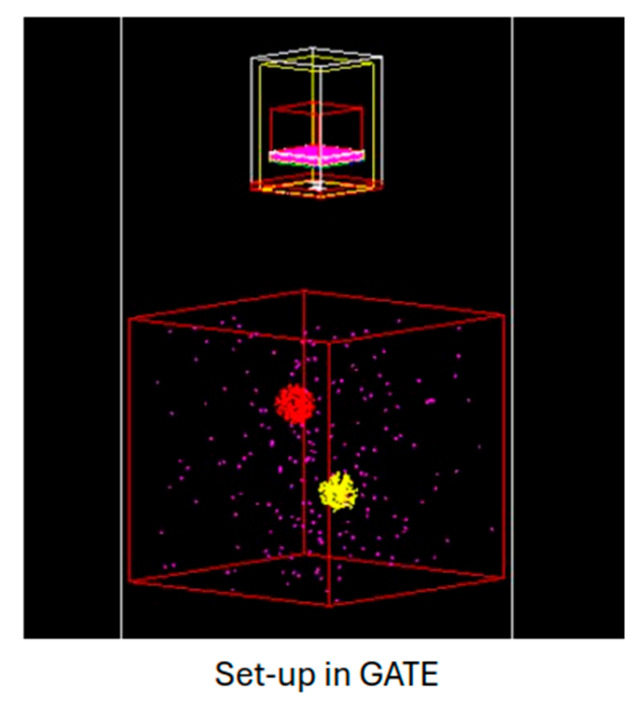
Model of the 2 spheres in GATE toolkit, at 100 mm (red sphere) and at 125 mm (yellow sphere) distance from the pinhole collimator.

Additionally, we investigated the impact on image deformation when the source-to-collimator distance changes due to the different minification factor. For this purpose, a water box was simulated 10 cm away from the camera, filled with very low activity as background radiation (37 MBq), which is not expected to contribute to the noise of the image. Inside the box, which had dimensions of 200 × 200 × 200 mm^3^, two identical spheres with a diameter of 12.4 mm were placed at different depths (100 and 125 mm from the pinhole collimator) and offsets (as illustrated in Figure 3) and were filled with 370 MBq. For validation purposes, the same measurement was performed experimentally with the developed gamma camera. Using this setup, we measured the activity within a region of interest (ROI) located at the center of each sphere and then calculated the activity ratio between the two spheres (furthest to closest one) to compare our results for both cases (simulation and experiment). We additionally compared our findings with the “theoretical” ratio based on geometric parameters.

#### 2.3.4. Preclinical Phantom Study

**Figure 4:**
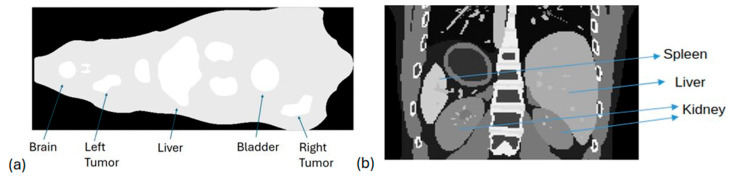
(**a**) Central slice of 3D rat phantom used for both MC simulations and real experiments; (**b**) part of the XCAT phantom which was used in GATE MC simulations.

Moreover, we investigated the feasibility of separating neighboring organs with equal activity levels through simulations and experiments using a rat phantom for preclinical applications. More specifically, a 3D fillable rat phantom with dimensions of 265 × 171 × 751 mm^3^ was used in digital form for GATE simulations (with a pixel size at 0.2 × 0.2 × 0.2 mm^3^) and after 3D printing for the actual experiments [39] (physical phantom). The bottom surface of the phantom in both cases was set 100 mm away from the pinhole collimator. We performed a study with activities in different organs of interest to validate the separation of neighboring organs comparable to the system’s resolution. We set equal activities of 18.5 MBq in the left tumor, liver, bladder, and right tumor, where the left tumor–liver distance was 17 mm and the bladder–right tumor distance was 9 mm. The number of primaries was equal to ~6 × 10^10^. Figure 4a depicts the rat phantom used for both simulations and actual experiments.

#### 2.3.5. Clinical Simulations

Following the preclinical simulations, we executed simulations on clinical data to assess the reliability of our system for real clinical examinations. Initially, we used the XCAT anthropomorphic phantom, which is a voxelized hybrid phantom designed to accurately model the intricate shapes of real human organs. By assigning a specific material to each voxel of the phantom based on its corresponding density, XCAT can accurately represent the attenuation map of the human body, mimicking its anatomical composition. During this study, a total activity of 6560 MBq, derived from a clinical examination was distributed into the phantom. More precisely, 75% of ^177^Lu activity was assigned to the kidneys, 15% to the liver, 7.5% to the spleen, and 2.5% to the remaining organs. The distance between the XCAT’s surface and the pinhole collimator was 10 cm, while the time acquisition of the simulation was 6.8 s (to have ~4.5 × 10^10^ simulated primaries). Figure 4b illustrates the XCAT phantom used in our study, where the main organs are depicted.

As a final clinical simulation, we used a clinical SPECT/CT image (^177^Lu acquisition), which was incorporated in GATE to reproduce a real clinical acquisition with the use of the portable imaging device. The clinical data used in the current study originated from the Centre Léon-Bérard (Lyon, France) [41]. The ^177^Lu biodistribution was defined by the SPECT image, which acted as an activity map, while the attenuation map was derived from the CT image. The surface of the patient was also placed 10 cm from the pinhole collimator. In this scenario, the activity of a specific organ was compared to the activity in the background of each method to assess the efficiency of our camera.

## 3. Results

### 3.1. In Silico and Experimental Investigation of the System

The simulated outputs were evaluated and compared with experimental investigations following the design and construction of the actual portable gamma camera. Subsequently, both the simulated and experimental results for all respective geometrical phantoms are presented.

#### 3.1.1. Non-Uniformity Correction with Flood Source

**Figure 5:**
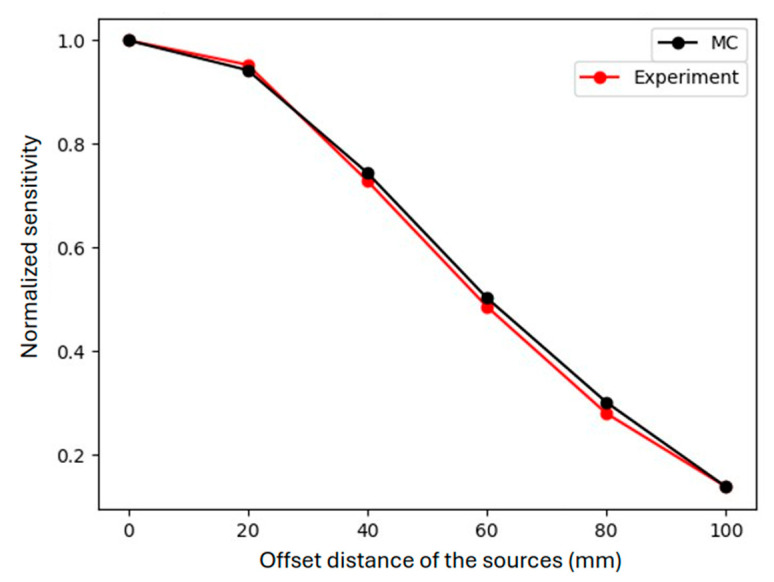
Normalized detected counts for MC simulations and actual experimental measurements of ^177^Lu sources placed on different offset distances from the center of FOV (zero offset).

The need for uniformity correction of the acquired images is demonstrated by the off-center measurements of ^177^Lu point sources (described in Section 2.3.3) both experimentally and with MC simulations, as seen in Figure 5, where detected counts are plotted over the corresponding offset distances of source locations. An identical decrease in detected counts for offset distances beyond 20 mm is observed for experimental and simulated data.

To rectify these irregularities in the acquired images, in both cases (simulations and actual experiments), the flood source images were used to restore the activity’s uniformity, as described in Section 2.3.1.

**Figure 6:**
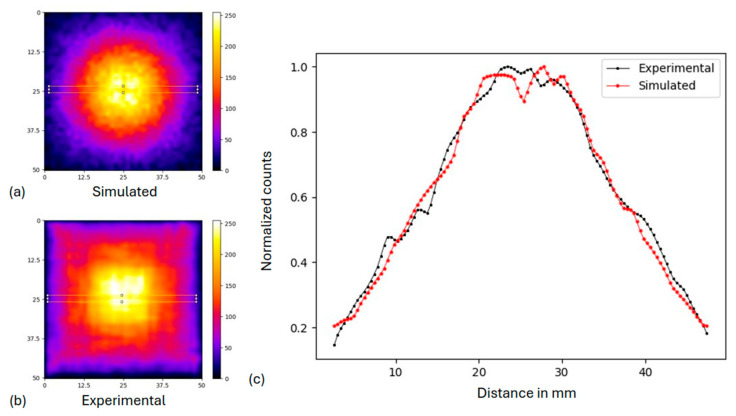
Simulated (**a**) and experimental (**b**) flood source images. Size scale of images is in mm. (**c**) Normalized profile comparison of the two flood sources in the selected area.

Figure 6 depicts the acquired flood source images for both the simulated (a) and experimental data (b). A normalized profile comparison is also presented for both cases, as extracted from the selected areas (highlighted in yellow) of central slices, additionally highlighting the image non-uniformity. The effect of higher concentrated activity in the center of the FOV is similar in both cases, decreasing the uniformity across the detector. The uncertainty of this non-uniformity measurement was determined to be 33% and 21% for the experimental and the simulated case, respectively (as the ratio of standard deviation to the mean value of the profiles, as is typical for such detector evaluation [45]). These images are to be used for the uniformity correction for each case.

#### 3.1.2. Derenzo-like Phantom

**Figure 7:**
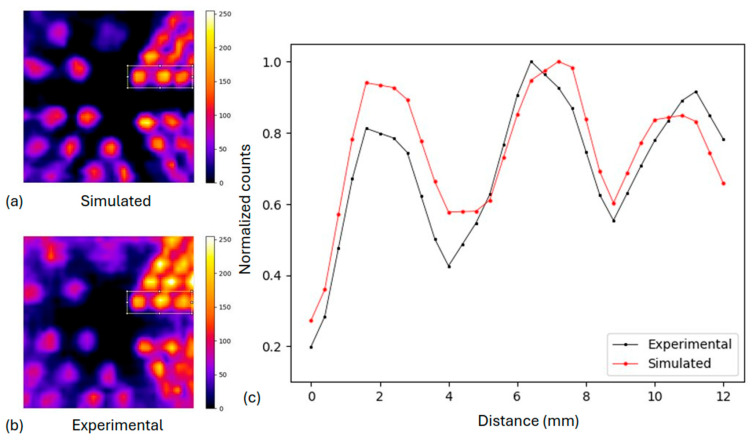
(**a**) Simulated Derenzo phantom with uncertainty of 3%, (**b**) experimental Derenzo phantom with uncertainty of 5%. In both images, flood correction has been applied. (**c**) Normalized profile comparison of the selected area of rod diameter of 1.0 cm.

The comparison between the simulated Derenzo-like phantom and the corresponding experimental image is presented in Figure 7, after applying the flood correction in both cases. The corresponding normalized profiles (Figure 7c) illustrate the activity distribution across the rods with a diameter of 1.0 cm (as presented in Figure 2) at the highlighted areas, where the peaks from the corresponding rods can still be distinguished in both cases. The spatial resolution is therefore determined to be 1 cm by analyzing all the profiles. The experimental output presents almost identical spatial resolution compared to the simulation.

#### 3.1.3. Off-Center Point Sources and Spheres Sources at Different Depths

**Figure 8:**
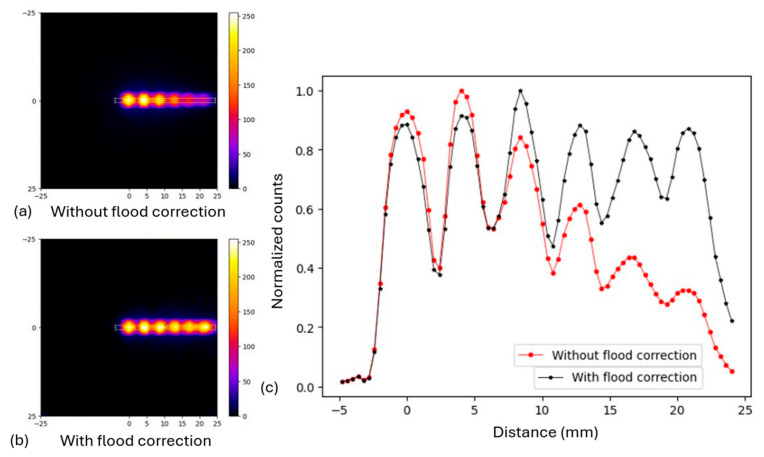
Simulated outputs for the 6 off-center point sources with statistical uncertainty of 3%, merged in one image, (**a**) without flood correction, (**b**) with flood correction (distance values from the image center in mm), and (**c**) the respective normalized profiles of the merged images on the highlighted areas.

Six (6) point sources were positioned at different horizontal positions from the pinhole collimator (as described in Section 2.3.3). Figure 8 presents the merged outputs of MC simulations in one image, with and without the flood correction, together with their normalized profiles (measured in the central orthogonal ROIs, as highlighted on the merged images). One can see that the detector’s sensitivity without the flood correction decreased along the horizontal offset from the center, as already seen in Figure 5, while it remained at the same level after the flood correction. This highlights the successful effect of flood correction in achieving a uniform distribution of the activity across the final image.

The system’s sensitivity was calculated to be 5.2 ± 0.4 Cps/MBq and 6.5 ± 0.8 Cps/MBq for the simulated and experimental cases, respectively. It should be noted that the experimental sensitivity was determined with a higher uncertainty (13%) compared to the simulated value (8%). Therefore, one should not conclude that the experimental sensitivity is definitively higher. The simulated and experimental values agree well within their respective uncertainties.

**Figure 9:**
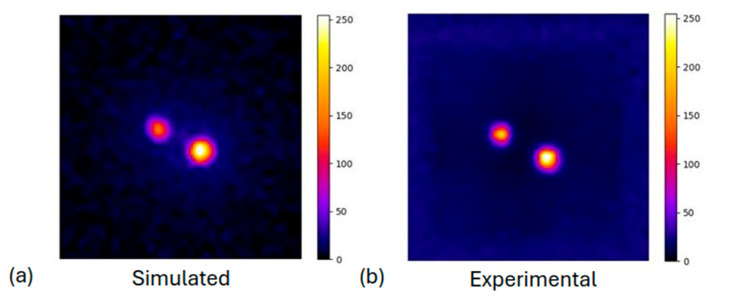
(**a**) Simulated and (**b**) experimental sphere phantom images at different depths.

Regarding the effects of object minification, the two sphere sources were positioned at different depths (see Section 2.3.3) from the pinhole collimator. Figure 9 shows the resulting images, both the simulated and the experimental one, alongside the simulation model of the setup in the GATE environment. The applied flood correction corresponds to the distance of 100 mm, where we optimized our detection system. In addition, the activity ratio of the two spheres, namely the detected counts from the furthest sphere (at 125 mm) to the counts of the closest one (at 100 mm), was measured to be 0.74 in simulations and 0.63 in experiments, respectively. The uncertainties of the calculations were lower than 5% and 3% for the experimental and the simulated data, respectively. Based purely on geometrical factors related to distance and the difference in solid angle—as governed by the inverse square law—the sensitivity drop is estimated to be a factor of 0.64. This estimate aligns more closely with the experimental ratio. However, for accurate theoretical calculations, it is important to note that the geometrical factors in a pinhole detection system are more complex. As shown by calculated ratios, the sensitivity is reduced by a factor of 1.35 and 1.59 for the simulation and the experiment, respectively, when the distance between the two spheres is 25 mm. This effect is also important as it is combined with additional minification of the object (1:6.25 at distance 125 mm), compared to the reference distance of 100 mm (1:5 scale).

### 3.2. Preclinical Study: Rat Phantom

**Figure 10:**
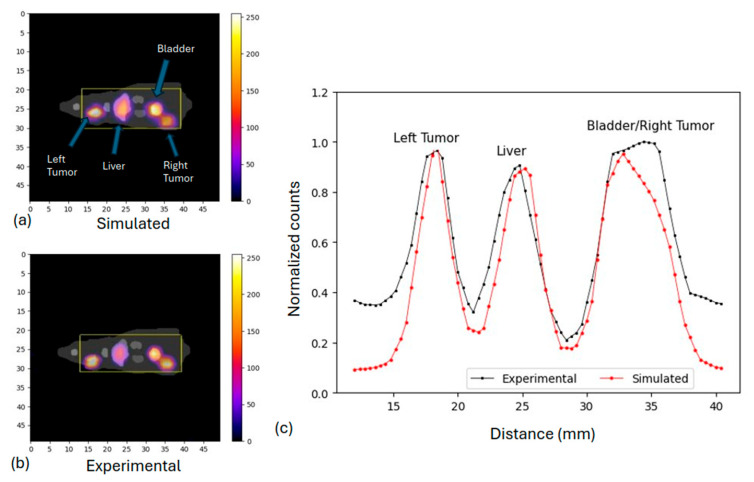
(**a**) Simulated rat phantom with 3% uncertainty; (**b**) experimental rat phantom with 5% uncertainty. Size of images is in mm; (**c**) normalized profiles within the selected area.

Both the MC simulation and the actual experiment on the 3D rat phantom with activity on the liver, bladder, and two tumors (left and right, adjacent to the bladder at 9 mm), as illustrated in Figure 10a,b, showed that the right tumor and bladder were still well distinguishable, as seen in Figure 10c. It is also shown that this is at the limit of the resolution, which was found to be 1 cm.

### 3.3. System Evaluation in Clinical Scenarios

**Figure 11:**
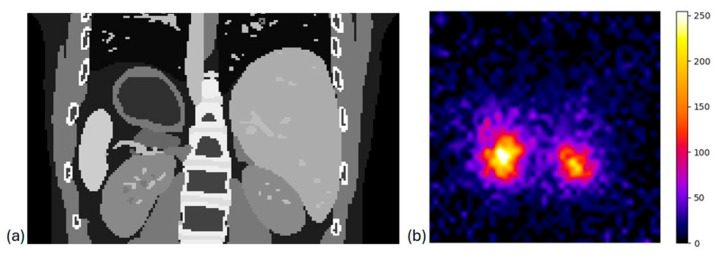
(**a**) Anatomical information from the XCAT phantom (coronal view) and (**b**) the simulated output of XCAT model in coronal view, with ^177^Lu distributed 75% to kidneys, 15% to liver, 7.5% to spleen, and 2.5% to rest organs.

**Figure 12:**
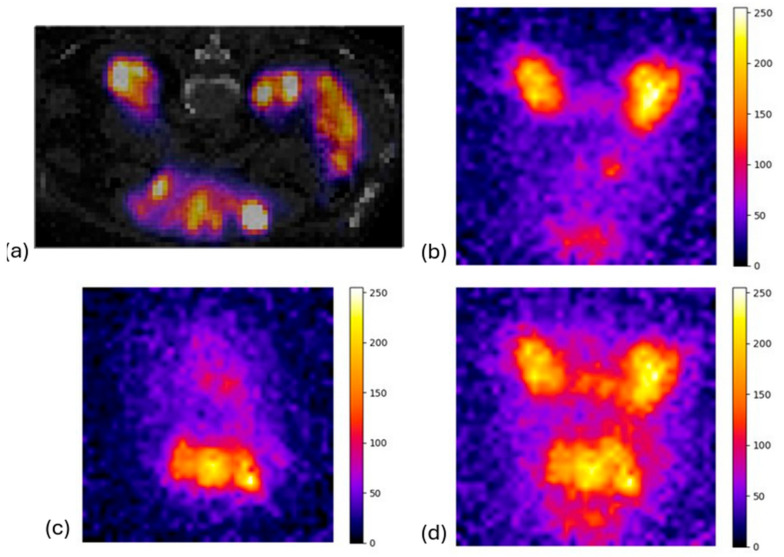
(**a**) Clinical SPECT/CT ^177^Lu axial image used as source in GATE; (**b**) anterior and (**c**) posterior output of the MC simulation, respectively; and (**d**) merged simulated image of anterior and posterior view.

Regarding the study using the XCAT anthropomorphic model phantom, to assess the performance of our portable pinhole gamma camera in a clinical scenario, Figure 11 shows that kidneys, where the highest activity concentration was present, were clearly observed according to the expected minification factor. This result is expected since the pinhole collimator in our camera caused minification of the images and aligned with our goal of obtaining a larger FOV within the captured images and hence a larger area of the patient. An identical behavior was observed when we simulated a clinical scenario (using real SPECT/CT data in GATE) as illustrated in Figure 12.

To validate the accuracy and effectiveness of our imaging system for clinical applications, we calculated the simulated activity in the background and in the left kidney, compared to the activity of the right kidney, as seen in Table 2. The computed ratio on the clinical SPECT image (which is referred to as the ratio on clinical data in Table 2) was performed in an ROI selected in the merged slices of SPECT (2D image). Considering the deformation of the organs due to the different distance from the camera (due to the depth of the patient), we repeated the simulation, capturing both the anterior and posterior planar images. The merged output of the two images is also presented in Figure 12d to highlight the impact of the source-to-collimator distance for different structures. The uncertainties for all cases were lower than 5%.

## 4. Discussion

The clinical application of ^177^Lu has significantly advanced the field of theranostics, particularly through its integration into targeted radioligand therapy [46]. Its dual emission properties—therapeutic beta particles and diagnostic gamma rays—make it uniquely suited for both diagnosis and post-therapy monitoring, enabling researchers to noninvasively assess biodistribution, dosimetry, and tumor uptake [47]. This imaging approach provides essential insights into pharmacokinetics and target specificity, facilitating early optimization of therapeutic agents and improving the prediction of treatment efficacy and safety.

In this study, the design and evaluation of a pinhole collimator in a portable gamma camera is presented, involving a comprehensive approach combining in silico data through MC simulations and experimental ones, allowing the optimization of the design parameters for enhanced image quality and sensitivity. The GATE MC toolkit was utilized to investigate the optimal parameters of the system prior to its development. The proposed system was developed using a tungsten pinhole collimator achieving a spatial resolution equal to 1.0 cm with a 1:5 minification when the object-to-pinhole distance was 10 cm. Such a spatial resolution is considered appropriate for ^177^Lu post-therapy imaging, as it can clearly distinguish small objects and accurately visualize the biodistribution of the therapeutic radionuclide within the target tissue. Both simulations and real experiments of the geometrical phantoms helped to evaluate the resolution, sensitivity, and overall performance of the system. Moreover, two additional spheres were used to investigate the effect of the minification and the difference in the sensitivity due to the different distances of the objects from the collimator. Our study showcased a high level of agreement between the MC simulations and the experimental results, validating the accuracy of the modeled configuration of the portable system as well as its performance.

It is worth mentioning that differences were also observed in the two setups (simulated and real detector), which are mainly due to the physical characteristics of the camera. Such visual difference is depicted in the two cases of the flood source image (Section 3.1.1), where the experimental visualization is depicted as a more “squared” image compared to the simulated one. The position of an event computation is affected by the discretized geometry of the photodetector, the readout circuit encoding, and the inter-crystal scattering effect, which are highly observed in the small crystal elements size [48]. Thus, a more “squared” distribution, like the actual geometry of the detector, is observed. However, one can observe the angle-dependent sensitivity effect also in the experimental setup. Non-uniformity corrections are typically performed to compensate for such issues related to different detector geometries and electronics. In our case, the performed flood correction compensates for the angle-dependent sensitivity effect both in the simulation and experimental results, leading to semi-quantitative information in the final images.

Overall, the percentage differences between the actual and measured activity ratio present lower values at the GATE simulations in contrast to the experimental data. MC simulations provide ideal situations and a consistent framework for assessing the accuracy of the activity ratios, while the experimental setup involves inaccuracies, such as the positioning of sources, equipment calibration, and/or manufacturing failures that affect the accuracy of the measurements.

As far as the clinical-wise study using the XCAT anthropomorphic model is concerned, the effect of the increased FOV of 25 × 25 cm^2^ in contrast to our 5 × 5 cm^2^ knife-edge aperture gamma camera was observed (at an object–collimator distance of 10 cm for 1:5 minification). The altered FOV limits the spatial coverage of the camera, requiring careful positioning and scanning techniques to capture the desired anatomical structures accurately. By opting for a pinhole collimator with minification, we aimed to maximize the actual FOV. This decision was driven by the aim of capturing as much anatomical information as possible within the constraints of a portable system that can be easily moved within a hospital.

Several limitations need to be mentioned, as the proposed system incorporates inhomogeneities and deformations of the objects/phantoms. The proposed prototype of the portable gamma camera incorporates one single pinhole collimator, which provides quite limited sensitivity for fast post-therapy imaging due to the restricted number of photons accepted through the single aperture. This constraint becomes particularly significant when imaging radionuclides with a medium–low photon yield or experiments with low statistics. The radioactivity values chosen for this study are high due to the limitation that the sensitivity of the system is low (based on one single pinhole). In contrast, a multi-pinhole collimator design can significantly enhance sensitivity by allowing simultaneous acquisition of projections through multiple apertures. This increases the total number of detected photons without compromising spatial resolution, especially when the pinholes are strategically placed to avoid overlapping projections. Previous studies have demonstrated that multi-pinhole SPECT systems can achieve higher sensitivity and an improved signal-to-noise ratio compared to single-pinhole configurations, making them suitable for dynamic or low-activity imaging tasks [49]. Future upgrades of this study will incorporate the design of a multi-pinhole collimator since the increase in sensitivity is of high importance. The attenuation correction and the minification factors of different depths should be also investigated to optimize both quantification and the imaging.

While patient-specific dosimetry often requires co-registered anatomical imaging, the proposed camera is not intended for that purpose in its current form. Instead, it is designed as a practical, portable tool for fast bedside imaging to qualitatively assess ^177^Lu biodistribution, detect unexpected uptake, or monitor relative changes across time points. Its use is particularly valuable in settings where full SPECT/CT is unavailable or impractical, offering clinicians an accessible means to support treatment monitoring and decision-making. Overall, this study has provided valuable insights into the performance of our portable pinhole camera in a clinical setting. The intentional minification achieved through the chosen pinhole collimator has allowed us to observe a larger FOV while still maintaining improved spatial resolution. Future improvements, including multi-pinhole configurations, anatomical co-registration strategies, and depth-dependent correction algorithms, are expected to enhance image quality and localization accuracy, thereby facilitating more detailed analysis and interpretation of the acquired SPECT images.

## 5. Conclusions

In conclusion, the comparison study between MC simulations and experimental evaluations of a portable SPECT pinhole collimator camera for resolution and sensitivity assessment has demonstrated promising results. MC simulations provided a robust computational framework for modeling the imaging system, accurately simulating the intricate physics processes involved in photon interactions and detector responses. Through these simulations, the camera’s parameters were optimized and fine-tuned to ensure optimal performance. Complementing the simulations, experimental evaluations played a vital role in validating the simulation results and assessing the camera’s real-world capabilities. The comparison between the Monte Carlo simulations and experimental results demonstrated a high level of agreement, affirming the reliability and accuracy of the simulation model.

This comparison study highlights the importance of integrating Monte Carlo simulations with real-world experimental evaluations to assess and validate the pinhole-based system’s performance. The successful implementation of this prototype approach encourages the development of a reliable and effective tool for clinicians from diagnosis until the acquisition of high-quality post-therapy images of ^177^Lu at different time points. In peptide receptor radionuclide therapy (PRRT), the administered activity is commonly fixed empirically and is suboptimal, which leads to large variability between patients in the absorbed dose to organs at risk and tumors. Such a portable device aims to provide clinicians with an assistant tool capable of capturing additional images between the therapy cycles, enabling them to monitor treatment progress and optimize therapeutic strategies more effectively. The usefulness of an optimized portable detection system could be further used for the personalization of the administered activity during PRRT. This study reflects the results of an innovative prototype portable system for ^177^Lu applications. Further investigation is needed to study a multi-pinhole concept as well as to develop correction algorithms for the deformation of the objects towards an approach with great promise in enhancing patient care and improving outcomes in ^177^Lu therapy.

## Figures and Tables

**Table 1:** Detector structure, materials, and technical characteristics.

Tungsten shielding box	
Thickness	5 mm
Outer dimensions	70 × 70 × 100 mm^3^
Pinhole collimator	
Diameter (d)	1.2 mm
Acceptance angle (a)	94 degrees
Channel height	1 mm
Scintillator	
Ce pixelated array	50 × 50 mm^2^ GAGG
Crystal dimensions	1 × 1 × 5 mm^3^ (0.2 mm septa)
Optical guide (glass) thickness	1 mm
PSPMTs	
Dimensions	48.5 × 48.5 × 32.5 mm^3^ each
Peak QE (@ 380 nm)	10%
Model	H12700A Hamamatsu
FPGA-based data acquisition system	

**Table 2:** Counts ratio between kidneys.

Organ at SPECT Image	Ratio on Simulated Data	Ratio on Clinical Data
Left kidney/right kidney	0.81	0.77
Background/right kidney	0.09	0.1

## Data Availability

The data presented in this study are available on request from the corresponding author due to restrictions imposed by the company that owns the data, as they contain proprietary information and are under commercial confidentiality.

## References

[1] Strosberg J., El-Haddad G., Wolin E., Hendifar A., Yao J., Chasen B., Mittra E., Kunz P.L., Kulke M.H., Jacene H. (2017). Phase 3 Trial of ^177^Lu-Dotatate for Midgut Neuroendocrine Tumors. N. Engl. J. Med..

[2] Paluri R.K., Killeen R.B. (2024). Neuroendocrine Tumor Lu-177-Dotatate Therapy. StatPearls.

[3] Fu J., Qiu F., Stolniceanu C.R., Yu F., Zang S., Xiang Y., Huang Y., Matovic M., Stefanescu C., Tang Q. (2022). Combined use of ^177^Lu-DOTATATE peptide receptor radionuclide therapy and fluzoparib for treatment of well-differentiated neuroendocrine tumors: A preclinical study. J. Neuroendocrinol..

[4] Sartor O., de Bono J., Chi K.N., Fizazi K., Herrmann K., Rahbar K., Tagawa S.T., Nordquist L.T., Vaishampayan N., El-Haddad G. (2021). Lutetium-177-PSMA-617 for Metastatic Castration-Resistant Prostate Cancer. N. Engl. J. Med..

[5] Garje R., Rumble R.B., Parikh R.A. (2022). Systemic Therapy Update on ^177^Lutetium-PSMA-617 for Metastatic Castration-Resistant Prostate Cancer: ASCO Rapid Recommendation. J. Clin. Oncol..

[6] Garin E., Tselikas L., Guiu B., Chalaye J., Edeline J., Assenat E., Tacher V., Terroir-Cassou-Mounat M., Mariano-Goulart D., Amaddeo G. (2021). Personalised versus standard dosimetry approach of selective internal radiation therapy in patients with locally advanced hepatocellular carcinoma (DOSISPHERE-01): A randomised, multicentre, open-label phase 2 trial. Lancet Gastroenterol. Hepatol..

[7] Danieli R., Milano A., Gallo S., Veronese I., Lascialfari A., Indovina L., Botta F., Ferrari M., Cicchetti A., Raspanti D. (2022). Personalized dosimetry in targeted radiation therapy: A look to methods, tools and critical aspects. J. Pers. Med..

[8] Danieli R., Stella M., Leube J., Tran-Gia J., Marin C., Uribe C.F., Vanderlinden B., Reynaert N., Flamen P., Levillain H. (2023). Quantitative ^177^Lu SPECT/CT imaging for personalized dosimetry using a ring-shaped CZT-based camera. EJNMMI Phys..

[9] Mu’minah I.A.S., Hidayati N.R., Widodo P., Shintawati R., Soejoko D.S. (2020). Investigation of Image Quality for Quantitative Lu-177 in SPECT imaging: A Phantom Study. J. Phys. Conf. Ser..

[10] Sagisaka Y., Takahashi Y., Hosokawa S., Kanazawa N., Yamamoto H., Takai G., Nagano K. (2024). Acquisition Conditions for Lu-177 DOTATATE Imaging. Radiation.

[11] Huizing D.M.V., Sinaasappel M., Dekker M.C., Stokkel M.P.M., de Wit-van der Veen B.J. (2020). 177 Lutetium SPECT/CT: Evaluation of collimator, photopeak and scatter correction. J. Appl. Clin. Med. Phys..

[12] Toderas L., Chen D., Iravani A., Scherzer Z., Erickson M., Miyaoka R. (2023). Clinical Implementation of Lu-177 PSMA therapy, including SPECT/CT imaging post therapy. J. Nucl. Med..

[13] Peters S.M.B., Mink M.C.T., Privé B.M., de Bakker M., de Lange F., Muselaers C.H.J., Mehra N., Witjes J.A., Gotthardt M., Nagarajah J. (2023). Optimization of the radiation dosimetry protocol in Lutetium-177-PSMA therapy: Toward clinical implementation. EJNMMI Res..

[14] Roth D., Larsson E., Sundlöv A., Sjögreen Gleisner K. (2020). Characterisation of a hand-held CZT-based gamma camera for ^177^Lu imaging. EJNMMI Phys..

[15] Aguiar P., Silva-Rodríguez J., Herranz M., Ruibal A. (2014). Preliminary experience with small animal SPECT imaging on clinical gamma cameras. Biomed. Res. Int..

[16] Bugby S.L., Farnworth A.L., Brooks W.R., Perkins A.C. (2024). Seracam: Characterisation of a new small field of view hybrid gamma camera for nuclear medicine. EJNMMI Phys..

[17] D’Arienzo M., Cozzella M., Fazio A., Ungania S., Cazzato M., Iaccarino G., D’Andrea M., Strigari L., Fenwick A., Cox M. (2016). Absolute gamma camera calibration for quantitative SPECT imaging with ^177^Lu. Phys. Medica.

[18] Raskin S., Gamliel D., Abookasis D., Ben-Haim S., Chicheportiche A. (2023). Towards accurate ^177^Lu SPECT activity quantification and standardization using lesion-to-background voxel ratio. EJNMMI Phys..

[19] Weber D.A., Ivanovic M., Franceschi D., Strand S.E., Erlandsson K., Franceschi M., Atkins H.L., Coderre J.A., Susskind H., Button T. (1994). Pinhole SPECT: An approach to in vivo high resolution SPECT imaging in small laboratory animals. J. Nucl. Med..

[20] Van Audenhaege K., Van Holen R., Vandenberghe S., Vanhove C., Metzler S.D., Moore S.C. (2015). Review of SPECT collimator selection, optimization, and fabrication for clinical and preclinical imaging. Med. Phys..

[21] Vastenhouw B., Beekman F. (2007). Submillimeter total-body murine imaging with U-SPECT-I. J. Nucl. Med..

[22] Huang W., Mok G.S.P. (2023). Multi-pinhole collimator design in different numbers of projections for brain SPECT. Front. Med..

[23] Ilisie V., Moliner L., Morera C., Nuyts J., Benlloch J.M. (2021). Gamma Camera Imaging with Rotating Multi-Pinhole Collimator. A Monte Carlo Feasibility Study. Sensors.

[24] Jiang N., Liu H., Xue M., Li C., Gao L., Liu F., Wu J., Liu Y. (2024). Performance evaluation of a novel multi-pinhole SPECT system. Nucl. Instrum. Methods Phys. Res. Sect. A Accel. Spectrometers Detect. Assoc. Equip..

[25] Islamian J.P., Azazrm A., Mahmoudian B., Gharapapagh E. (2015). Advances in pinhole and multi-pinhole collimators for single photon emission computed tomography imaging. World J. Nucl. Med..

[26] Cha H., Cho K., Jung Y.-J., Bae S., Kim K.M., Kim M., Lee H., Lee K. (2021). Development of organ-specific dual-head single-photon emission computed tomography using variable pinhole collimator. Nucl. Instrum. Methods Phys. Res. Sect. A Accel. Spectrometers Detect. Assoc. Equip..

[27] Gonzalez-Montoro A., Vera-Donoso C.D., Konstantinou G., Sopena P., Martinez M., Ortiz J.B., Carles M., Benlloch J.M., Gonzalez A.J. (2022). Nuclear-medicine probes: Where we are and where we are going. Med. Phys..

[28] Jan S., Santin G., Strul D., Staelens S., Assié K., Autret D., Avner S., Barbier R., Bardiès M., Bloomfield P.M. (2004). GATE: A simulation toolkit for PET and SPECT. Phys. Med. Biol..

[29] Jan S., Benoit D., Becheva E., Carlier T., Cassol F., Descourt P., Frisson T., Grevillot L., Guigues L., Maigne L. (2011). GATE V6: A major enhancement of the GATE simulation platform enabling modelling of CT and radiotherapy. Phys. Med. Biol..

[30] Sarrut D., Arbor N., Baudier T., Borys D., Etxebeste A., Fuchs H., Gajewski J., Grevillot L., Jan S., Kagadis G.C. (2022). The OpenGATE ecosystem for Monte Carlo simulation in medical physics. Phys. Med. Biol..

[31] Sarrut D., Bardiès M., Boussion N., Freud N., Jan S., Létang J.-M., Loudos G., Maigne L., Marcatili S., Mauxion T. (2014). A review of the use and potential of the GATE Monte Carlo simulation code for radiation therapy and dosimetry applications. Med. Phys..

[32] Sarrut D., Bała M., Bardiès M., Bert J., Chauvin M., Chatzipapas K., Dupont M., Etxebeste A., Fanchon L.M., Jan S. (2021). Advanced Monte Carlo simulations of emission tomography imaging systems with GATE. Phys. Med. Biol..

[33] Allison J., Amako K., Apostolakis J., Arce P., Asai M., Aso T., Bagli E., Bagulya A., Banerjee S., Barrand G. (2016). Recent Developments in Geant4. Nucl. Instrum. Meth..

[34] Weldstone Advanced Solutions. https://www.weldstone.com/en.

[35] Epic-Crystals. https://www.epic-crystal.com/.

[36] Hamamatsu. https://www.hamamatsu.com.

[37] Fysikopoulos E., Loudos G., Georgiou M., David S., Matsopoulos G. (2012). A Spartan 6 FPGA-based data acquisition system for dedicated imagers in nuclear medicine. Meas. Sci. Technol..

[38] Smith M.F., Jaszczak R.J. (1997). The effect of gamma ray penetration on angle-dependent sensitivity for pinhole collimation in nuclear medicine. Med. Phys..

[39] BIOEMTECH. https://bioemtech.com/product/phantom/.

[40] Segars W.P., Sturgeon G., Mendonca S., Grimes J., Tsui B.M. (2010). 4D XCAT phantom for multimodality imaging research. Med. Phys..

[41] Plachouris D., Eleftheriadis V., Nanos T., Papathanasiou N., Sarrut D., Papadimitroulas P., Savvidis G., Vergnaud L., Salvadori J., Imperiale A. (2023). A radiomic- and dosiomic-based machine learning regression model for pretreatment planning in ^177^Lu-DOTATATE therapy. Med. Phys..

[42] Cox B.L., Graves S.A., Farhoud M., Barnhart T.E., Jeffery J.J., Eliceiri K.W., Nickles R.J. (2016). Development of a novel linearly-filled Derenzo microPET phantom. Am. J. Nucl. Med. Mol. Imaging.

[43] Hoffmann J.V., Janssen J.P., Kanno T., Shibutani T., Onoguchi M., Lapa C., Grunz J.-P., Buck A.K., Higuchi T. (2020). Performance evaluation of fifth-generation ultra-high-resolution SPECT system with two stationary detectors and multi-pinhole imaging. EJNMMI Phys..

[44] Higaki Y., Kobayashi M., Uehara T., Hanaoka H., Arano Y., Kawai K. (2013). Appropriate collimators in a small animal SPECT scanner with CZT detector. Ann. Nucl. Med..

[45] Bhatia B.S., Bugby S.L., Lees J.E., Perkins A.C. (2015). A scheme for assessing the performance characteristics of small field-of-view gamma cameras. Phys. Medica Eur. J. Med. Phys..

[46] Dash A., Pillai M.R.A., Knapp F.F. (2015). ^177^Lu radiopharmaceuticals for therapy and diagnosis: A review. J. Radioanal. Nucl. Chem..

[47] Banerjee S., Pillai M.R.A., Ramamoorthy N. (2005). Therapeutic applications of ^177^Lu and radio-lanthanides in nuclear medicine. Appl. Radiat. Isot..

[48] Lamprou E., Sanchez F., Benlloch J., Gonzalez A. (2020). In-depth evaluation of TOF-PET detectors based on crystal arrays and the TOFPET2 ASIC. Nucl. Instrum. Methods Phys. Res. Sect. A Accel. Spectrometers Detect. Assoc. Equip..

[49] van der Have F., Vastenhouw B., Ramakers R.M., Branderhorst W., Krah J.O., Ji C., Staelens S.G., Beekman F.J. (2009). U-SPECT-II: An ultra-high-resolution device for molecular small-animal imaging. J. Nucl. Med..

